# Optical Characteristic Research on Fiber Bragg Gratings Utilizing Finite Element and Eigenmode Expansion Methods

**DOI:** 10.3390/s140610876

**Published:** 2014-06-19

**Authors:** Yuejing He, Xuanyang Chen

**Affiliations:** Electronic Engineering Department, National Chin-Yi University of Technology, Taichung 41170, Taiwan; E-Mail: s4a113002@student.ncut.edu.tw

**Keywords:** fiber Bragg grating, finite element method, eigenmode expansion method

## Abstract

Compared with coupled-mode theory (CMT), which is widely used for studies involving optical fiber Bragg gratings (FBGs), the proposed investigation scheme is visualized, diagrammatic, and simple. This method combines the finite element method (FEM) and eigenmode expansion method (EEM). The function of the FEM is to calculate all guided modes that match the boundary conditions of optical fiber waveguides. Moreover, the FEM is used for implementing power propagation for HE_11_ in optical fiber devices. How the periodic characteristic of FBG causes this novel scheme to be substantially superior to CMT is explained in detail. Regarding current numerical calculation techniques, the scheme proposed in this paper is the only method capable of the 3D design and analysis of large periodic components. Additionally, unlike CMT, in which deviations exist between the designed wavelength *λ_D_* and the maximal reflection wavelength *λ*_max_, the proposed method performs rapid scans of the periods of optical FBG. Therefore, once the operating wavelength is set for the component design, the maximal reflection wavelength of the final products can be accurately limited to that of the original design, such as *λ* = 1550 nm. Furthermore, a comparison between the period scan plot and the optical spectra plot for FBG indicated an inverse relationship between the periods and wavelengths. Consequently, this property can be used to predict the final FBG spectra before implementing time-consuming calculations. By employing this novel investigation scheme involving a rigorous design procedure, the graphical and simple calculation method reduces the studying time and professional expertise required for researching and applying optical FBG.

## 1. Introduction

In recent decades, optical fiber Bragg gratings (FGBs) have been developed into essential devices with numerous applications for optical fiber telecommunication and small sensor systems. These applications include wavelength-division multiplexer (WDM) systems [1–3], optical add/drop multiplexer (OADM) devices [4–6], multichannel dispersion compensators [7,8], and surface plasmon resonance (or localized surface plasmon resonance) optical fiber sensors [9–15]. FBGs can also function as mirrors [16], incorporating a copropagating HE_11_, which is coupled to a counterpropagating mode of an identical type, and a loss filter [17], in which the leakage wave away from the optical fiber can be effectively suppressed.

Thus far, the most widely used method in research on the properties and applications of FBG is the coupled-mode theory (CMT). For scholars beginning to study FBGs, learning CMT is crucial for mastering the physical concepts of FBGs and understanding their properties. Using the following three aspects, how CMT was applied for implementing FBG analysis and design is explained [18–20].

### 1.1. Defining the Optical Fiber Structure and Calculating the Solution of Maxwell's Equations

**Figure 1.**
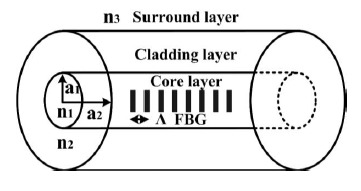
The geometric configuration of a communication optical fiber with FBG.

The geometric configuration of a communication optical fiber consists of three layers: the core, cladding, and surrounding. The radii and refractive index of these layers are a_1_ = 2.5 μm, a_2_ = 62.5 μm, n_1_ = 1.458, n_2_ = 1.45, and n_3_ = 1, as shown in Figure 1. Theoretically, the dimension of the surrounding layer can be regarded as infinite. The dispersion relation equations can be derived from the Maxwell equations and the boundary conditions of the geometric configuration of an optical fiber cross-section are rendered. Thereafter, all guided modes that can exist in the optical fiber are acquired. The corresponding effective refractive index $n_{\text{eff}}^{v}$ of each guided mode is determined by solving the dispersion relation equations [13,18–20]. From a purely mathematical perspective, guided modes are solutions to differential equations that satisfy certain boundary conditions. Unless otherwise indicated, this study focused on analyzing single-mode communication fibers.

### 1.2. Defining the Mathematical Model of FBG and Explaining the Coupled-Mode Equation

For FBG production, a single-mode communication optical fiber containing photosensitive materials in the core layer was used. Subsequently, the phase mask obtaining specific period was irradiated using ultraviolet light to create constructive and destructive interference on the single-mode optical fiber, thereby changing the refractive index of the core layer. For uniform FBG, the mathematical equation can be expressed as follows [9–13,18]:

(1) $$n_{1}=n_{1}+n_{1}\sigma \left[1+\cos(\frac{2\pi }{{\text{Λ}}_{\text{FBG}}}z)\right]$$

where *δ n* = *n*_1_ *σ* is the peak-induced index change and Λ*_FBG_* is the period of FBG. From a mathematical viewpoint, all optical fiber guided modes are mutually orthogonal before FBG appears. The power is not exchanged (or coupled) between any two guided modes throughout the propagation process. Nevertheless, while FBG exists in the core layer, it induces power perturbation among the modes. The appropriately designed period Λ*_FBG_* can destroy the orthogonality among the guided modes and achieve power coupling. Finally, under the condition of *δ n* ≪ *n*_1_, the mathematical model of mode coupling is as follows [9–13,18]:

(2) $$\frac{dA_{\mu }}{dz}=iA_{v}K_{v-\mu }\text{exp}\left[i\left(\beta_{v}-\beta_{\mu }\right)z\right]$$

with:

(3) $$K_{v-\mu }=\frac{w}{2}\frac{\text{Δ}ɛ\int_{A\infty }\left(E_{r}^{v}E_{r}^{\mu }+E_{\varphi }^{v}E_{\varphi }^{\mu }\right)dA-\frac{\text{Δ}ɛ}{\text{Δ}ɛ+ɛ}\int_{A\infty }\left(E_{z}^{v}E_{z}^{\mu }\right)dA}{\int_{0}^{2\pi }\int_{0}^{\infty }\left(E_{r}^{\mu }H_{\varphi }^{\mu }-E_{\varphi }^{\mu }H_{r}^{\mu }\right)dA}$$

where *A_u_* and *A_v_* are the amplitude for the *μth* and *vth* guided modes in the optical fiber, respectively; *β_μ_* and *β_v_* are the propagation constants for the *μth* and *vth* guided modes, respectively; *K_v_*_−_*_μ_* is the coupling coefficient between the *μth* mode and *vth* mode; Δ*ε* is the amount of change in permittivity caused by FBG in the core layer; and *w* is the angular frequency.

### 1.3. Defining the Bar and Cross Transmission Power for the Optical Spectra

To research and calculate the spectral properties of FBG, the mathematical equation of the bar transmission power and cross transmission power are defined as follows [10,13,18]:

(4) $$t_{=}=\frac{{\left|R\left(z\right)\right|}^{2}}{{\left|R\left(0\right)\right|}^{2}}$$

and:

(5) $$t_{\times }=\frac{{\left|S\left(z\right)\right|}^{2}}{{\left|R\left(0\right)\right|}^{2}}$$

where |*R*(*z*)|^2^ is the power of HE_11_ and |*S*(*z*)|^2^ is the power of the cladding mode. In other words, the bar transmission power is defined as the power ratio of HE_11_ after propagation to length (*z*) to its power at input end (*z* = 0). Similarly, the power ratio of the new mode to the power of HE_11_ at input end (*z* = 0) is defined as cross transmission power if HE_11_ is coupled to another cladding mode following its propagation to length (*z*). The optical spectra of FBG can be acquired by calculating and plotting the relationship between the bar transmission power (or the cross transmission power) and the wavelength.

In this section, minimal mathematical equations were used to provide comprehensive, yet simple, explanations of the physical concepts in CMT. CMT is mathematically challenging for FBG beginners and extremely difficult for application designers. Accordingly, this study proffered a visualized and simple calculation method featuring a combination of two widely known numerical simulation methods. A rigorous design procedure was incorporated into this novel method to reduce the studying time and professional expertise required for researching and applying FBG.

The remainder of this paper is organized as follows: Section 2 provides a simple description of the theory related to the finite element method (FEM). Regarding numerical methods, the cutting precision of triangular elements inevitably affects the validity of the obtained guided modes. Based on the mathematical concept, the orthogonality between any two solutions must be 0 if all of the obtained solutions are accurate. Consequently, in this study, a reverse-thinking method was adopted to obtain the acceptable orthogonality, which was 10^−4^ (10×log_10_(10^−4^) = −40 dB), based on adjusting the cutting resolution and implementing simulations [9–12,18].

Section 3 introduces the eigenmode expansion method (EEM). A specification is provided to interpret in detail how EEM causes guided modes to propagate in FBG optical fibers. Additionally, how this novel technique is beneficial to CMT is explained. Inevitably, EEM also causes accuracy problems. The numerical method enables optical wave propagations, primarily through a Fourier series transform. Consequently, despite being in nonabsorbing media, the overall power decreases as the propagation length increases once the number of guided modes adopted during the propagation is inadequate. Accordingly, this study involved using a reverse-thinking method and testing using various guided modes for propagation to examine power loss during this procedure. Subsequently, the appropriate number of guided modes adopted could be determined [9–12,18].

Section 4 summarizes the content of Sections 2 and 3, proposes a strict, simple, and integrated design procedure, reports FBG analyses, and describes the procedure. Furthermore, numerous plots were employed to express the calculation results and facilitate studying CMT, which features equations and abstract concepts.

Finally, Section 5 concludes the paper. The proposed novel numerical technique is summarized and the results acquired in Section 4 are used to prove its applicability for designing sizeable and periodic devices. This consequently reduces the studying time and professional expertise required for researching and applying FBG.

## 2. The FEM

The FEM is a numerical calculation technique that has been used extensively to resolve partial differential equations that match specific boundary conditions. The variational principle, domain cutting, and interpolation function are the core principles of this technique. FEM employs a variation algorithm to transfer the primal problem into functional extreme value problems, which differ from the original problem but yield equivalent results. Two distinct mathematical explanations for the identical physical problem exist. The interpolation function and the cut domain were applied to convert the problem in which the functional solved for the minimum value into a set of multiple linear algebraic equations after determining the functional extreme value problem. The solutions for the primal problem are acquired by resolving these linear algebraic equations [9–12,18,21].

In this study, a triangular element was used to cut the geometric region of the *x*-*y* plane of the optical fiber. A high cutting resolution results in precise calculation results; however, this procedure involves substantially more simulation time. Therefore, appropriate discretization of the target solution domain is vital when applying FEM. As previously mentioned, all guided modes must be mutually orthogonal. The orthogonality can be represented using the following Equation (6) in [18]. Completing total simulation requires an extremely large memory capacity and long mode solution time. To match the computational capacity of a laboratory server, the orthogonal values of the obtained guided modes were reviewed and used to regulate the resolution of triangular cutting suitably. Unless otherwise indicated, the cutting resolutions used in this study satisfied the criterion of the orthogonal values being smaller than 10^−4^ (10 × log_10_(10^−4^) = −40 dB). To satisfy this guideline, the minimum number of elements in the core layer was approximately 100, and the minimum number of elements in the cladding layer was approximately 2500. Following cutting, all guided modes in the optical fiber at an operating wavelength of *λ* = 1550 nm could be resolved. In a single-mode communication optical fiber, only one core mode, HE_11_, with an effective refractive index of $n_{\text{eff}}^{\text{core}}\left(n_{2}<n_{\text{eff}}^{\text{core}}<n_{1}\right)$, and numerous cladding modes, with an effective refractive index of $n_{\text{eff}}^{\text{cladding}}\left(n_{3}<n_{\text{eff}}^{\text{cladding}}<n_{2}\right)$, could be acquired.

**Figure 2.**
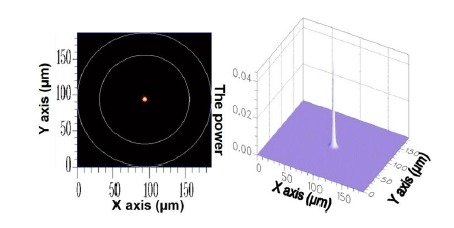
The 2D and 3D power diagram of HE_11_ with an effective refractive index of $n_{\text{eff}}^{\text{core}}=1.451975$.

As previously mentioned, the cross-section of the optical fiber grating used in this study should be uniform. Consequently, the mode coupling phenomenon appeared between only HE_11_ and the cladding modes of azimuthal order *l* = 1, whereas HE_11_ inputs the FBG domain. Therefore, only the solution of cladding modes where *l* = 1 was investigated. The core mode HE_11_ is acquired using FEM; its 2D and 3D power distributions are shown in Figure 2.

**Figure 3.**
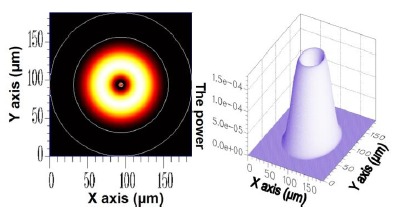
The 2D and 3D power diagram of the cladding mode (*ν* = 1) with an effective refractive index of $n_{\text{eff}}^{v=1}=1.449947$.

**Figure 4.**
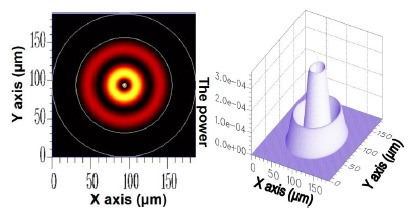
The 3D power diagram of the cladding mode (*ν* = 3) with an effective refractive index of $n_{\text{eff}}^{v=3}=1.449769$.

**Figure 5.**
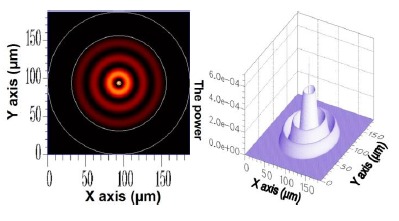
The 2D and 3D power diagram of the cladding mode (*ν* = 5) with an effective refractive index of $n_{\text{eff}}^{v=5}=1.449470$.

**Figure 6.**
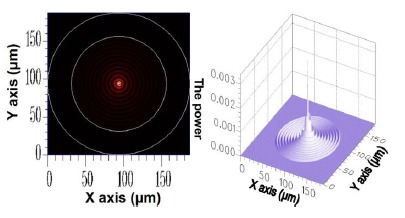
The 2D and 3D power diagram of the cladding mode (*ν* = 21) with an effective refractive index of $n_{\text{eff}}^{v=21}=1.443007$.

**Figure 7.**
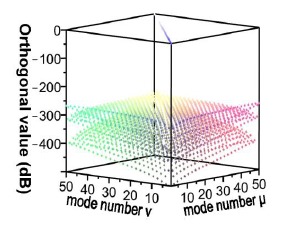
Plot of the orthogonal values for the 50 guided modes.

For the *v* = 1, *v* = 3, *v* = 5, and *v* = 21 cladding modes, the 2D and 3D power distributions are shown in Figures 3, 4, 5 and 6, respectively. Initially, the 50 resolved guided modes consisted of one HE_11_ and 49 cladding modes, where *v* = 1 – 49 and *l* = 1. The orthogonal values between these 50 modes were calculated and tested and the results are shown in Figure 7. Each mode possessed a self-orthogonal value of 1 (10 × log_10_(1) = 0 dB) and the orthogonal values between two distinct guided modes complied with the criterion; in other words, they were smaller than 10^−4^ (10 × log_10_(10^−4^) = −40 dB).

## 3. The EEM

**Figure 8.**
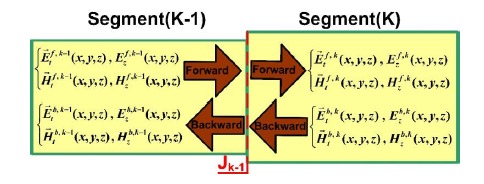
Fourier series expansion for the copropagation and counterpropagation modes between the two adjacent uniform segment objects, Segment (K-1) and Segment (K).

In a periodical waveguide structure, the first period of the waveguide is intercepted first. All of the uniform waveguides that formed during the first period were referred to as segments. The primary function of the EEM is to completely propagate (*i.e.*, yielding no loss) the energies of the previous uniform waveguide Segment (K-1) to the next uniform waveguide Segment (K) (Figure 8) [9–12,22–24]. Fourier series expansion is used to expand a known function by using multiple base functions. In other words, Fourier series expansion refers to converting known functions into the frequency domain. Similarly, the EEM uses multiple modes (*i.e.*, eigenmode or eigenvector) to expand a known optical wave; in other words, convert known optical waves into the eigenvalue domain. Therefore, the term “expansion” in both EEM and Fourier series expansion is functionally identical. Hence, the mode coefficients of the EEM can be easily obtained by following the procedures for solving Fourier coefficients. The electromagnetic fields in Segment (K-1) can be presented as follows [12]:

(6) $${\vec{E}}_{t}^{k-1}\left(x,y,z\right)=\sum_{\ell =1}^{L}\left(C_{\ell }^{f,k-1}e^{i\beta_{\ell }^{k-1}z}+C_{\ell }^{b,k-1}e^{-i\beta_{\ell }^{k-1}z}\right){\vec{e}}_{t\ell }^{k-1}\left(x,y\right)$$

(7) $$E_{z}^{k-1}\left(x,y,z\right)=\frac{\nabla_{t}\times {\vec{H}}_{t}^{k-1}}{-iw{ɛ}_{k-1}}$$

(8) $${\vec{H}}_{t}^{k-1}\left(x,y,z\right)=\sum_{\ell =1}^{L}\left(C_{\ell }^{f,k-1}e^{i\beta_{\ell }^{k-1}z}-C_{\ell }^{b,k-1}e^{-i\beta_{\ell }^{k-1}z}\right){\vec{h}}_{t\ell }^{k-1}\left(x,y\right)$$

(9) $$H_{z}^{k-1}\left(x,y,z\right)=\frac{\nabla_{t}\times {\vec{E}}_{t}^{k-1}}{iw\mu }$$

where *L* represents the total number of modes used in Segment (K-1) and $C_{\ell }^{f,k-1}$ and $C_{\ell }^{b,k-1}$ represent the coefficients of copropagating and counterpropagating modes, respectively. Similarly, the electromagnetic fields in Segment (K) can be expressed as follows [12]:

(10) $${\vec{E}}_{t}^{k}\left(x,y,z\right)=\sum_{m=1}^{M}\left(C_{m}^{f,k}e^{i\beta_{m}^{k}z}+C_{m}^{b,k}e^{-i\beta_{m}^{k}z}\right){\vec{e}}_{tm}^{k}\left(x,y\right)$$

(11) $$E_{z}^{k}\left(x,y,z\right)=\frac{\nabla_{t}\times {\vec{H}}_{t}^{k}}{-iw{ɛ}_{k}}$$

(12) $${\vec{H}}_{t}^{k}\left(x,y,z\right)=\sum_{m=1}^{M}\left(C_{m}^{f,k}e^{i\beta_{m}^{k}z}-C_{m}^{b,k}e^{-i\beta_{m}^{k}z}\right){\vec{h}}_{tm}^{k}\left(x,y\right)$$

(13) $$H_{z}^{k}\left(x,y,z\right)=\frac{\nabla_{t}\times {\vec{E}}_{t}^{k}}{iw\mu }$$

where *M* represents the total number of modes used in Segment (K) and $C_{\ell }^{f,k}$ and $C_{\ell }^{b,k}$ represent the copropagating and counterpropagating mode coefficients, respectively. These mode coefficients (*i.e.*, $C_{\ell }^{f,k}$ and $C_{\ell }^{b,k}$) are obtained using the orthogonal value of modes (e.g., the derivation principles of Equation (6) and Fourier coefficients). The corresponding equations are as follows [12]:

(14) $$C_{m}^{f,k}=\sum_{\ell =1}^{\ell }C_{n}^{f,k-1}e^{i\left(\beta_{\ell }^{k-1}-\beta_{m}^{k}\right)z}\left[\frac{\langle {\vec{e}}_{t\ell }^{k-1},{\vec{h}}_{tm}^{k}\rangle +\langle {\vec{e}}_{tm}^{k},{\vec{h}}_{t\ell }^{k-1}\rangle }{\langle {\vec{e}}_{tm}^{k},{\vec{h}}_{tm}^{k}\rangle }\right]+\sum_{\ell =1}^{L}C_{n}^{b,k-1}e^{-i\left(\beta_{\ell }^{k-1}+\beta_{m}^{k}\right)z}\left[\frac{\langle {\vec{e}}_{t\ell }^{k-1},{\vec{h}}_{tm}^{k}\rangle -\langle {\vec{e}}_{tm}^{k},{\vec{h}}_{t\ell }^{k-1}\rangle }{\langle {\vec{e}}_{tm}^{k},{\vec{h}}_{tm}^{k}\rangle }\right]$$

(15) $$C_{m}^{b,k}=\sum_{\ell =1}^{L}C_{n}^{f,k-1}e^{i\left(\beta_{\ell }^{k-1}+\beta_{m}^{k}\right)z}\left[\frac{\langle {\vec{e}}_{t\ell }^{k-1},{\vec{h}}_{tm}^{k}\rangle -\langle {\vec{e}}_{tm}^{k},{\vec{h}}_{t\ell }^{k-1}\rangle }{\langle {\vec{e}}_{tm}^{k},{\vec{h}}_{tm}^{k}\rangle }\right]+\sum_{\ell =1}^{L}C_{n}^{b,k-1}e^{-i\left(\beta_{\ell }^{k-1}-\beta_{m}^{k}\right)z}\left[\frac{\langle {\vec{e}}_{t\ell }^{k-1},{\vec{h}}_{tm}^{k}\rangle +\langle {\vec{e}}_{tm}^{k},{\vec{h}}_{t\ell }^{k-1}\rangle }{\langle {\vec{e}}_{tm}^{k},{\vec{h}}_{tm}^{k}\rangle }\right]$$

where:

$$\langle \vec{e},\vec{h}\rangle =\int_{A\infty }\vec{e}\times \vec{h}•ẑdA$$

The *L* modes used in Segment (K-1) were distinct from the *M* modes used in Segment (K). In other words, for a periodical waveguide structure, FEM was executed for all of the uniform waveguides (*i.e.*, segments) of the first period to solve the modes of the segments. Assuming that a periodical waveguide had a total number of *P* periods, computing the first period nearly equaled completing a simulation of the entire periodical waveguide. This characteristic indicated that combining FEM and EEM can substantially reduce the time and memory size required for a periodical waveguide simulation.

From a mathematical perspective, the base functions used in a Fourier series expansion must be complete, implying that all of the base functions must be included; otherwise, errors would be generated between the original function *f*(*x*) and the Fourier series ∑*a_n_*sin(*w_n_x*) + *b_n_*cos(*w_n_x*) after Fourier series expansion. The corresponding equation is as follows [12]:

(16) $$\text{error}=\left|f\left(x\right)-\left[\sum a_{n}\sin\left(w_{n}x\right)+b_{n}\cos\left(w_{n}x\right)\right]\right|$$

**Figure 9.**
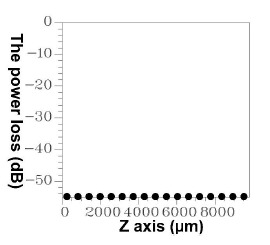
The relationships between the propagation length and the power loss for 50 guided modes.

Based on similar principles, errors occur in the EEM. In a numerical simulation, all of the modes could not be included in the computation because of the limited capability of the servers. Thus, errors cannot be prevented, but must be controlled at an acceptable level. Accordingly, through reverse thinking, an acceptable upper limit for power loss was defined (10^−4^). Before initiating component simulation, the EEM was applied to various numbers of modes and the power loss conditions were inspected. If the power loss values exceeded the upper limit, the number of modes was increased until the minimal number of modes that satisfied the criteria was determined. Figure 9 shows the power loss conditions when 50 modes were used in the three types of segments, indicating that a loss of less than 10^−4^ (10 × log_10_(10^−4^) = −40 dB) was attained using 50 modes. Hence, unless otherwise specified, the number of modes was set to 50 during the component design and simulation [9–12,18].

CMT is the most commonly used method in fiber component research. The derivation procedures for CMT are presented in the appendix of reference [12] to facilitate comparison of the differences between CMT and EEM during mathematical derivation. Based on these assertions, the findings indicate that EEM and CMT can be used to expand modes. Although the EEM was developed based on CMT, it considers both the features of periodical components and error mechanisms. In addition, the EEM in conjunction with FEM can be applied to investigate and develop periodical components with complex structures [12,18].

## 4. Design and Simulation

**Figure 10.**
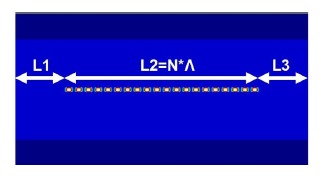
The *x*-*z* planar configuration of the optical fiber with FBG for numerical calculations.

Using the novel numerical calculation techniques described in Sections 2 and 3, a strict, simple, and complete design procedure was proposed for analyzing and designing optical FBG. This procedure involves the following six steps: (1) set relevant optical fiber and FBG parameters; (2) solve all of the guided modes by using the FEM; (3) test whether the orthogonal values of all of the guided modes satisfy the less than 10^−4^ (10 × log_10_(10^−4^) = −40 dB) requirement; (4) use the EEM to perform an algorithm for power propagation; (5) test whether the power loss obtained using the EEM satisfies the less than 10^−4^ (10 × log_10_(10^−4^) = −40 dB) requirement; (6) conduct FBG design and analysis. The geometric configuration of the optical fiber adopted for the calculations is shown in Figure 10, where L_1_ = 100 μm and L_3_ = 100 μm are ordinary single-mode optical fibers, whereas L_2_ = N*Λ (μm) is a single-mode FBG optical fiber.

**Figure 11.**
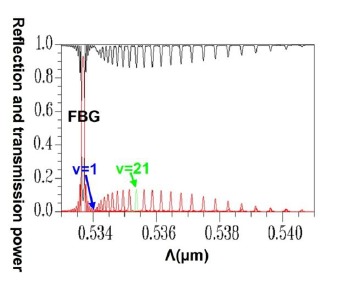
Plot between FBG period and the reflection (or transmission) power of the guided modes. The black line expresses the core mode and cladding modes transmission power. The red line expresses the core mode and cladding modes reflection power.

Applying a combination of the FEM and EEM, the relationship between the guided mode reflection (or transmission) power and the period of FBG can be rapidly scanned, as shown in Figure 11.

**Figure 12.**
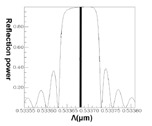
Plot between the FBG period and the reflection power for the total counterpropagating HE_11_ (core mode) with Λ = 0.5336803 μm.

**Figure 13.**
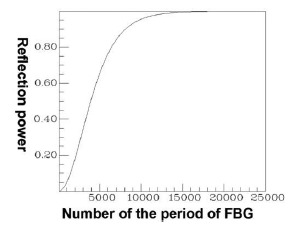
Plot between the FBG period number and the reflection power for the total counterpropagating HE_11_ (core mode) with N = 18,000 and Λ = 0.5336803 μm.

This provides crucial data regarding the period of FBG required for designing the counterpropagation of HE_11_, as well as the period of FBG required for coupling HE_11_ to a particular counterpropagating cladding mode. Figure 12 is a plot of the relationship between the total counterpropagating HE_11_ and the FBG period. Figure 13 is a diagram of the relationship between the total counterpropagating HE_11_ and the FBG period number. According to Figures 12 and 13, HE_11_ nearly achieves total counterpropagation of power when the FBG period is Λ = 0.5336803 μm and the FBG period number is N = 18,000.

**Figure 14.**
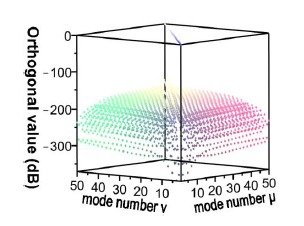
Plot of the power propagation in the *x*-*z* plane for the total counterpropagating HE_11_.

**Figure 15.**
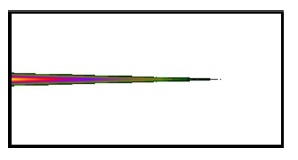
Plot between the orthogonal values of the 50 guided modes with N = 18,000, Λ = 0.5336803 μm, L = L_1_ + L_2_ + L_3_ = 9,806.2454 μm.

**Figure 16.**
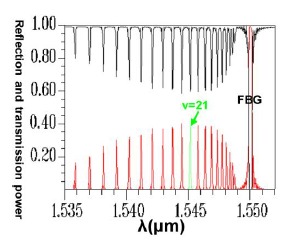
Plot between the propagation length and power loss for the total counterpropagating HE_11_ with N = 18,000, Λ = 0.5336803 μm, L = L_1_ + L_2_ + L_3_ = 9,806.2454 μm.

Using the FBG period and the FBG period number, HE_11_, was input from the left end of Figure 10, and the power propagation phenomenon of HE_11_ was inspected in the *x-z* plane (*y* = 0), as shown in Figure 14. HE_11_ evidently achieves total counterpropagation because of the interference of FBG. Additionally, to match the criterion for Step 3 of the design procedure, the orthogonal values in this example were tested, as shown in Figure 15. Each mode possessed a self-orthogonal value of 1 (10 × log_10_(1) = 0 dB) and the orthogonal values between two distinct guided modes complied with the criterion; in other words, they were smaller than 10^−4^ (10 × log_10_(10^−4^) = −40 dB). The propagation length and power loss of this example were also calculated, as shown in Figure 16. The power loss was evidently smaller than 10^−4^ (10 × log_10_(10^−4^) = −40 dB), which satisfied the criterion for Step 3 of the design procedure [9–12,18].

In CMT [19–20], numerous approximations and assumptions were employed during the derivation of equations to acquire exact solutions. These included δ *n* = *n*_1_ *σ* << *n*_1_, ignoring the longitudinal coupling coefficient $K_{v-\mu }^{z}$, and assuming that coupling occurs only between two distinct modes. Therefore, Equation (2) was simplified into two-mode coupled-mode equations. References [19] and [20] expressed that the designed wavelength of *λ_D_* shifts from the maximal reflection wavelength of *λ*_max_.

**Figure 17.**
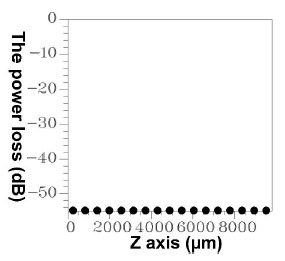
An optical spectra plot of the total counterpropagating HE_11_. The black line expresses the core mode and cladding modes transmission power. The red line expresses the core mode and cladding modes reflection power.

Conversely, following Step 1 in the design procedure, the operating wavelength is set at *λ_D_* = 1550 nm; all subsequent design steps are implemented using this wavelength. In Step 6, a propagation analysis of HE_11_ showed that the parameters required for the power to reach total counterpropagation were Λ = 0.5336803 μm and N = 18,000. According to these findings, the optical spectra plot was calculated as shown in Figure 17. This indicates that the maximum reflection wavelength *λ*_max_ is located exactly on *λ_D_* = 1550 nm.

**Figure 18.**
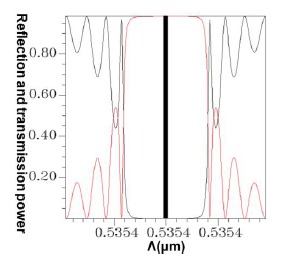
Plot between the FBG period and the reflection (or transmission) power for the total counterpropagating HE_11_. The black line expresses the core mode and cladding modes transmission power. The red line expresses the core mode and cladding modes reflection power.

Following a comparison between the optical spectra plot of the total counterpropagating HE_11_ (Figure 17) and the plot of the relationship between the FBG period and the reflection (or propagation) power for the total counterpropagating HE_11_ (Figure 18), the two plots exhibited inverse relationships between the coupling wavelength *λ* and FBG period Λ. Therefore, forecasting the final optical spectra of FBG from Figure 18 is possible before spending a substantial amount of time calculating FBG spectra. This concept can be interpreted using the following equation [19–20]:

(17) $$\lambda_{D}=\lambda_{\max}≅\left(n_{\text{eff}}^{\text{core}}+n_{\text{eff}}^{\text{cladding}-v}\right)\cdot {\text{Λ}}_{\text{FBG}}$$

In the optical spectra plot, the period Λ_FBG_ of FBG is constant; consequently, the positions of various cladding modes *v* in the optical spectra plot are *λ_D_* = *λ*_max_. These positions are proportional to the effective refractive indices $n_{\text{eff}}^{\text{cladding}-v}$ of the cladding modes. In the FBG spectra plot, when the mode order *v* increases, the position of the mode coupling *λ_D_* = *λ*_max_ shifts from right to left. In the period scan plots, the operating wavelength *λ_D_* = *λ*_max_ is constant; consequently, the positions where various cladding modes *v* appear in the period scan plot are inversely proportional to the effective refractive indices of the cladding modes $n_{\text{eff}}^{\text{cladding}-v}$. Accordingly, in the FBG period scan plot, when the mode order *v* increases, the position of the mode coupling shifts from left to right.

**Figure 19.**
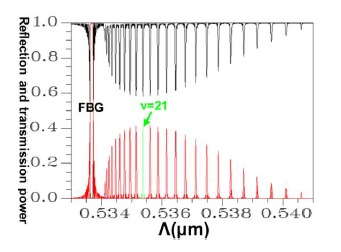
Plot between the FBG period and the reflection (or transmission) power for the total coupling from HE_11_ to a counterpropagating cladding mode (*ν* = 21) with Λ = 0.53537 μm. The black line expresses the core mode transmission power. The red line expresses the cladding mode (*ν* = 21) reflection power.

This explains why the FBG spectra scan plot has an inverse relationship with the coupling positions of all of the mode couplings in the period scan plot. Another design example was based on the coupling of HE_11_ and counterpropagating cladding modes *ν* = 21. Using the same analysis techniques, Figure 19 shows the relationship between the FBG period and the propagation (or reflection) power for the total coupling phenomenon from HE_11_ to a counterpropagating cladding mode *ν* = 21.

**Figure 20.**
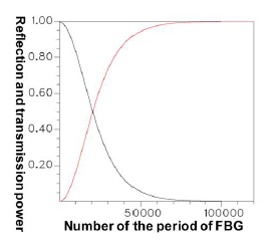
Plot between the FBG period number and the reflection (or transmission) power for the total coupling from HE_11_ to a counterpropagating cladding mode (*ν* = 21) with N = 120,000 and Λ = 0.53537 μm. The black line expresses the core mode transmission power. The red line expresses the cladding mode (*ν* = 21) reflection power.

**Figure 21.**
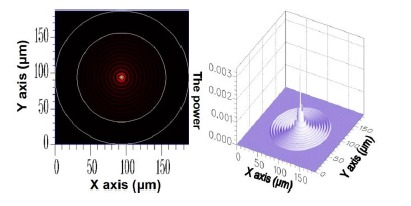
Plot of the power propagation in the *x*-*z* plane for the total coupling from HE_11_ to a counterpropagating cladding mode (*ν* = 21).

**Figure 22.**
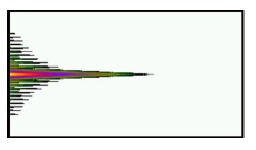
The 2D and 3D power plot of the cladding mode (*ν* = 21).

**Figure 23.**
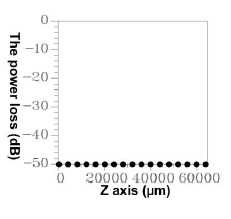
Plot between the orthogonal values of the 50 guided modes with N = 120,000, Λ = 0.53537 μm, L = L_1_ + L_2_ + L_3_ = 64,444.4 μm.

Figure 20 shows the relationship between the FBG period number and the propagation (or reflection) power for the total coupling phenomenon from HE_11_ to a counterpropagating cladding mode *ν* = 21. Figures 19 and 20 show that when period Λ = 0.53537 μm and the number of periods N = 120,000, HE_11_ is completely coupled to a counterpropagating cladding mode *ν* = 21. Additionally, using the period and the number of periods, HE_11_ was input from the left end of Figure 10 and the power propagation phenomenon of HE_11_ was inspected on the *x*-*z* plane (*y* = 0), as shown in Figure 21. The power of HE_11_ was completely coupled to a counterpropagating mode (*ν* = 21) during the propagation. To prove the precision of the coupling phenomenon, the 2D and 3D power plots (Figure 22) were dropped from the input terminal of Figure 21, which is on the left side. Comparisons between Figure 6 and Figure 22 illustrate that the two guided modes are identical, further proving the precision of the proposed technique. To match the requirements for Step 3 of the design procedure, the orthogonality of this sample was estimated and tested, as shown in Figure 23.

**Figure 24.**
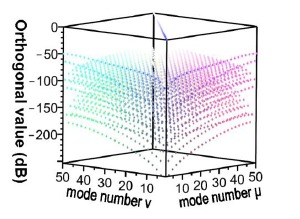
Plot between the propagation length and power loss for the total coupling from HE_11_ to a counterpropagating cladding mode (ν = 21) with N = 120,000, Λ = 0.53537 μm, L = L_1_ + L_2_ + L_3_ = 64,444.4 μm.

Each mode possessed a self-orthogonal value of 1 (10 × log_10_(1) = 0 dB) and the orthogonal values between two different guided modes complied with the criterion; in other words, they were smaller than 10^−4^ (10 × log_10_(10^−4^) = −40 dB) [9–12,18]. Additionally, the propagation length and power loss of this design sample were tested, as shown in Figure 24.

**Figure 25.**
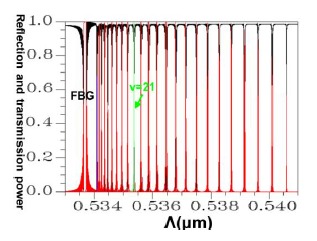
An optical spectra plot for the total coupling from HE_11_ to a counterpropagating cladding mode (*ν* = 21). The black line expresses the core mode and cladding modes transmission power. The red line expresses the core mode and cladding modes reflection power.

**Figure 26.**
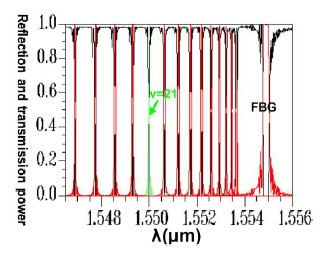
Plot between the FBG period and the reflection (or transmission) power for the total coupling from HE_11_ to a counterpropagating cladding mode (*ν* = 21). The black line expresses the core mode and cladding modes transmission power. The red line expresses the core mode and cladding modes reflection power.

The results indicated that the power loss satisfied the requirement of not exceeding 10^−4^ (10 × log_10_(10^−4^) = −40 dB), which is specifically for Step 5 of the design procedure [9–12,18]. Using the parameters Λ = 0.53537 μm and N = 120,000, the optical spectra can be calculated, as shown in Figure 25. The maximal reflection wavelength *λ*_max_ was again identical to the designed wavelength of *λ_D_* = 1550 nm. Additionally, identical to that for the counterpropagating HE_11_, the optical spectra of HE_11_, completely coupled to a counterpropagating cladding mode (*ν* = 21) as shown in Figure 25, were compared with the plot (Figure 26) of the relationship between the FBG period and the reflection (or transmission) power for the total coupling phenomenon from HE_11_ to a counterpropagating cladding mode (*ν* = 21). These two plots clearly exhibited a particular inverse relationship between coupling period and wavelength. Consequently, this property can be used to predict the final FBG spectra before implementing time-consuming calculations.

## 5. Conclusions

This paper proposed a visualized, diagrammatic, and simple numerical calculation technique. The proposed technique combines FEM and EEM with a strict, effortless, and integrated design procedure to perform FBG design and analyses. None of the existing numerical calculation techniques can be applied to 3D analysis and research of components comprising FBGs that are 1 to 7 cm in length. Section 3 explained that the application of the EEM consumes a relatively small amount of memory and time in the comprehensive analysis and design of large FBGs. The key step of the EEM is extracting single-period objects from periodic components and then recalculating them to obtain solutions. Section 3 established the uniqueness of this study. Additionally, Section 4 introduced one of the core techniques of this study. Numerous rigorous graphics presenting the results were provided, based on the design process, for enabling FBG novices and application designers entering the field of FBG to study CMT with the assistance of visualized graphics. This reduces the difficulty of directly studying equations and abstract mathematical models. Unlike the CMT, applying the proposed design procedure resulted in FBG end products that had a maximal reflection wavelength *λ*_max_ that precisely equaled the predesigned wavelength of *λ_D_*. A comparison between the period scan plot and the optical spectra plot for FBG indicated a certain inverse relationship between the periods and wavelengths. Consequently, this property can be used to predict the final FBG spectra before implementing time-consuming calculations. Additionally, power loss and the orthogonal values of the guided modes in the two examples were calculated and tested to ensure that the results complied with the requirements of the design process. This study used strict, simple, and graphical numerical techniques to facilitate understanding of the CMT and reduce the difficulty of studying FBG.
